# Circ_0030998 promotes tumor proliferation and angiogenesis by sponging miR-567 to regulate VEGFA in colorectal cancer

**DOI:** 10.1038/s41420-021-00544-7

**Published:** 2021-06-24

**Authors:** Longyang Jin, Chao Han, Tianyu Zhai, Xiaoyu Zhang, Chun Chen, Lei Lian

**Affiliations:** 1 Department of Colorectal Surgery, The Sixth Affiliated Hospital of Sun Yat-sen University, Guangzhou, Guangdong China; 2 Department of General Surgery, Shanghai General Hospital, Shanghai Jiaotong University School of Medicine, Shanghai, China; 3 Department of General Surgery, Xinhua Hospital, Shanghai Jiaotong University School of Medicine, Shanghai, China

**Keywords:** Oncogenes, miRNAs

## Abstract

Colorectal cancer (CRC) is one of the most common malignancies worldwide. Circular RNAs (circRNAs) are involved in pathological processes, especially in the development of cancers, but the roles of circRNAs in CRC are largely unknown. In this study, we investigated the role and underlying mechanisms of Circ_0030998 in CRC cell proliferation and angiogenesis. We found that Circ_0030998 was upregulated in CRC tissues and cells, and its upregulation was related to poor prognosis in CRC patients. Circ_0030998 promoted CRC cell proliferation in vitro and in vivo, and facilitated the angiogenesis of HUVECs. Mechanistic studies demonstrated that Circ_0030998 acted as a miR-567 sponge to relieve its inhibitory effect on VEGFA. Rescue assays validated that Circ_0030998 functioned in CRC cell proliferation and angiogenesis relying on VEGFA. Our findings clarified the Circ_0030998/miR-567/VEGFA regulation axis and indicated that Circ_0030998 could be a potential therapeutic target for CRC.

## Introduction

Colorectal cancer (CRC), one of the most common malignancies, has become the third leading cause of cancer-related deaths worldwide [1]. Even with the popularity of colonoscopy and the practice of advanced treatments including chemotherapy, radiotherapy, and immunotherapy, the overall survival of CRC patients has not increased significantly [2]. For patients of stage IV, the prognosis is extremely poor with a 5-year survival rate of less than 20% [3]. Although more and more oncogenes have been identified in the occurrence and development of CRC, the specific mechanisms are still obscure. So it’s urgently needed to explore the molecular mechanisms related to the progression of CRC.

Only about 1.5% of the human genome contains protein-coding genes, with the majority of the remaining transcribed into non-coding RNAs [4]. Accumulating studies have demonstrated that non-coding RNAs play important roles in the body’s growth, disease, aging, reproduction, and other physiological and pathological processes [5]. In terms of species complexity, non-coding genes may function more importantly than protein-coding genes [6]. Circular RNAs (circRNAs), characterized with a covalently closed loop, were first found in the RNA virus in 1970s [7], and were initially regarded as by-products of splicing from exons or introns of host genes [8]. However, more and more studies have shown that circRNAs are involved in pathological processes and could regulate gene expression in various ways [9]. CircRNAs could function as competing endogenous RNAs by sponging microRNAs to regulate gene expression. For example, circ_0020710 could promote melanoma cell proliferation, migration, and invasion by sponging miR-370-3p and then regulating the CXCL12 expression [10]. CircRNAs could also bind with RNA binding proteins (RBPs) directly, and affect the function of RBPs [11]. Some circRNAs could regulate the expression of host genes by interacting with RNA polymerase II [12]. A small part of circRNAs could even act as protein templates [13–15]. The covalently closed loop structure of circRNAs resulted in high stability, which helped them to be ideal biomarkers. Several circRNAs have been detected involved in the progression of colorectal cancer [16–18]. However, the identification of more functional circRNAs may facilitate the molecular-guided diagnosis and treatment of colorectal cancer.

In the present study, we identified a new circular RNA, Circ_0030998, that functioned as an oncogene in the proliferation and angiogenesis of CRC. Circ_0030998 was derived from the exon 3 of host gene LAMP1 and upregulated in CRC tissues and cell lines compared with nontumor tissues and non-tumorigenic colorectal epithelial cell line. High expression of Circ_0030998 was related to lymph node metastasis, TNM stage, and poor prognosis of CRC patients. Moreover, Circ_0030998 could promote CRC cell proliferation in vitro and in vivo. Mechanistically, Circ_0030998 acted as a miR-567 sponge to relieve the inhibitory effect of miR-567 on VEGFA, which played important roles in the proliferation and angiogenesis of CRC. In summary, the present study explored the mechanism of Circ_0030998 in the progression of CRC, demonstrated the Circ_0030998/miR-567/VEGFA axis, and provided a new potential target for the treatment against CRC.

## Results

### Identification of Circ_0030998

**Fig. 1:**
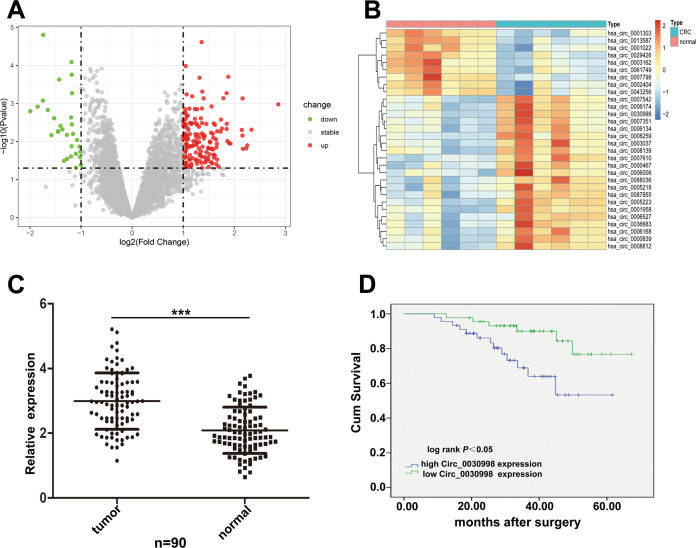
Circ_0030998 was upregulated in CRC tissues and associated with poor prognosis of CRC patients. **A** The difference of CircRNAs expression between CRC tissues and matched neighboring normal tissues exhibited by the volcano plots generated from microarray data GSE138589. **B** The heat map of the top 30 CircRNAs with the most significant differences from data GSE138589. **C** Relative expression of Circ_0030998 in CRC tissues and paired adjacent normal tissues (*n* = 90). **D** Correlation between Circ_0030998 expression and CRC patients’ survival. ***p < 0.001.

By analyzing the microarray data GSE138589, which compared six pairs of CRC tissues and matched neighboring normal tissues. A total of 155 upregulated circRNAs and 29 downregulated circRNAs with *p-*value < 0.05 and |fold change | > 2 were identified (Table S2). The differently expressed circRNAs were exhibited by volcano plots as shown in Fig. 1A. Among these 184 circRNAs, the top 30 ones (21 upregulated and 9 downregulated) with the most significant differences were selected for further study and shown by hierarchical clustering analysis in Fig. 1B. To screen out the circRNA that may be related to the progression of CRC cells, the expression of 21 upregulated circRNAs was tested in CRC tissues, and it was shown that Circ_0030998 had the highest level than other 20 circRNAs (data not shown). Therefore, we chose Circ_0030998 for further analysis.

According to the circBase (http://www.circbase.org/) and UCSC Genome Browser Home (http://genome.ucsc.edu/), we found that Circ_0030998 was 220 base pairs (bp) in length located at chr13:113963957-113964177 and it was derived from the exon 3 of host gene LAMP1, which acted as an oncogene in the progression of several cancers [19, 20].

### Circ_0030998 was significantly upregulated in CRC tissues and associated with a poor prognosis of CRC patients

Firstly, qRT-PCR was performed to identify the expression levels of Circ_0030998 in 90 pairs of CRC tissues and adjacent normal tissues. As shown in Fig. 1C, Circ_0030998 was significantly upregulated in CRC tissues compared to the adjacent normal tissues. Moreover, we divided 90 CRC patients into two groups according to the median level (cutoff value = 2.961) of the relative Circ_0030998 expression in tumor tissues: the high group (*n* = 45) and the low group (*n* = 45). Then Pearson chi-square tests were used to analyze the relationship between Circ_0030998 expression level and patients’ clinicopathologic features. It was demonstrated that high expression of Circ_0030998 was related to lymph node metastasis and TNM stage but not gender, age, tumor location, CEA level, and tumor size of CRC patients (Table 1).

**Table 1:** Correlation between Circ_0030998 expression and clinicopathologic features of CRC patients.

Characteristics	Case number	Circ_0030998 expression		*p*-Value
		High (*n* = 45)	Low (*n* = 45)	
Gender				0.515
Male	56	26	30	
Female	34	19	15	
Age				0.509
≤50	32	18	14	
>50	58	27	31	
Tumor Location				0.832
Colon	50	24	26	
Rectum	40	21	19	
CEA				0.673
≤10 ng/ml	43	23	20	
>10 ng/ml	47	22	25	
Tumor size				0.09
≤5 cm	49	20	29	
>5 cm	41	25	16	
Lymph node metastasis				0.032*
Yes	53	32	21	
No	37	13	24	
TNM stage				0.028*
I–II	33	11	22	
III–IV	57	34	23	

^*^p < 0.05.

Furthermore, Kaplan–Meier survival analysis was conducted to analyze the relationship between Circ_0030998 expression level and patients’ survival. It showed that patients with high Circ_0030998 levels had shorter survival compared to those with low levels (Fig. 1D). Univariate survival analysis showed that lymph node metastasis, TNM stage, and Circ_0030998 expression were prognostic factors. Multivariate Cox regression analysis demonstrated that only TNM stage and Circ_0030998 expression were independent prognostic factors for CRC patients (Table 2).

**Table 2:** Univariate and multivariate analysis of prognostic factors for overall survival in CRC patients.

Characteristics	Univariate analysis		multivariate analysis	
	HR	*P*-value	HR (95%CI)	*P*-value
Gender	0.02	0.888		
Age	0.022	0.883		
Tumor Location	2.427	0.119		
CEA	0.339	0.561		
Tumor size	0.942	0.332		
Lymph node metastasis	9.810	0.002^**^	3.738(0.811–17.235)	0.091
TNM stage	15.320	<0.001^***^	68.543(7.047–666.695)	<0.001^***^
Circ_0030998 expression	6.588	0.01^*^	0.224(0.074–0.682)	0.008^**^

^*^p < 0.05, **p < 0.01, *** *p* < 0.001.

### Circ_0030998 was upregulated in CRC cell lines and promoted tumor proliferation and angiogenesis in vitro

**Fig. 2:**
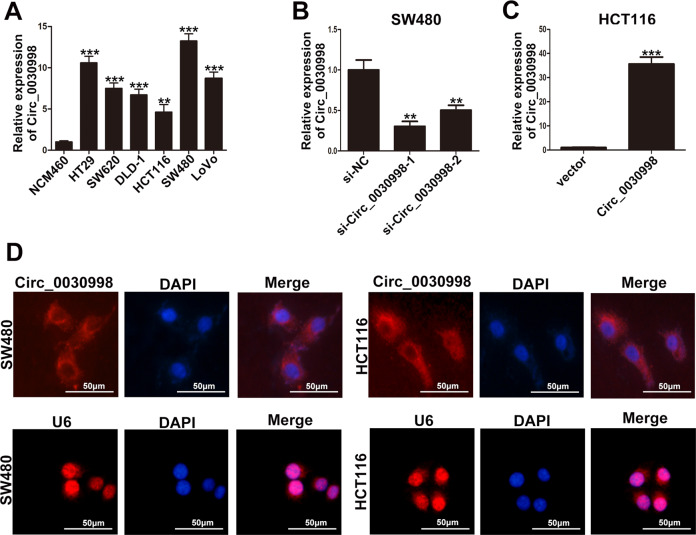
Circ_0030998 was upregulated in CRC cell lines and mainly localized in the cytoplasm of CRC cells. **A** Relative expression of Circ_0030998 in CRC cell lines and human non-tumorigenic colorectal epithelial cell line NCM460 determined by qRT-PCR. **B** The efficiency of siRNAs of Circ_0030998 in SW480 cells examined by qRT-PCR. **C** The efficiency of Circ_0030998 overexpression plasmid in HCT116 cells examined by qRT-PCR. **D** FISH images of Circ_0030998 expression in SW480 and HCT116 cells (red) (U6 as control). Nuclei were stained by DAPI (blue). **p < 0.01, ***p < 0.001.

The relationship of Circ_0030998 expression and prognosis of CRC patients suggested that Circ_0030998 was correlated with tumor malignancy. Thus, we investigated the functions of Circ_0030998 in CRC cell lines. Firstly, we examined the expression of Circ_0030998 in six CRC cell lines (HT29, SW620, DLD-1, HCT116, SW480, LoVo) and a human non-tumorigenic colorectal epithelial cell line (NCM460) by qRT-PCR. The results showed that Circ_0030998 was significantly upregulated in CRC cell lines than NCM460 (Fig. 2A). Then, the SW480 cell line which had the relatively highest expression of Circ_0030998, and the HCT116 cell line which had the relatively lowest expression of Circ_0030998 were chosen for further study. Two siRNAs targeting Circ_0030998 were transfected into SW480, and si-Circ_0030998-1 which was chosen for subsequent experiments exhibited better knockdown efficiency (Fig. 2B). Meanwhile, Circ_0030998 overexpression plasmid significantly upregulated the expression of Circ_0030998 in HCT116 cells (Fig. 2C).

**Fig. 3:**
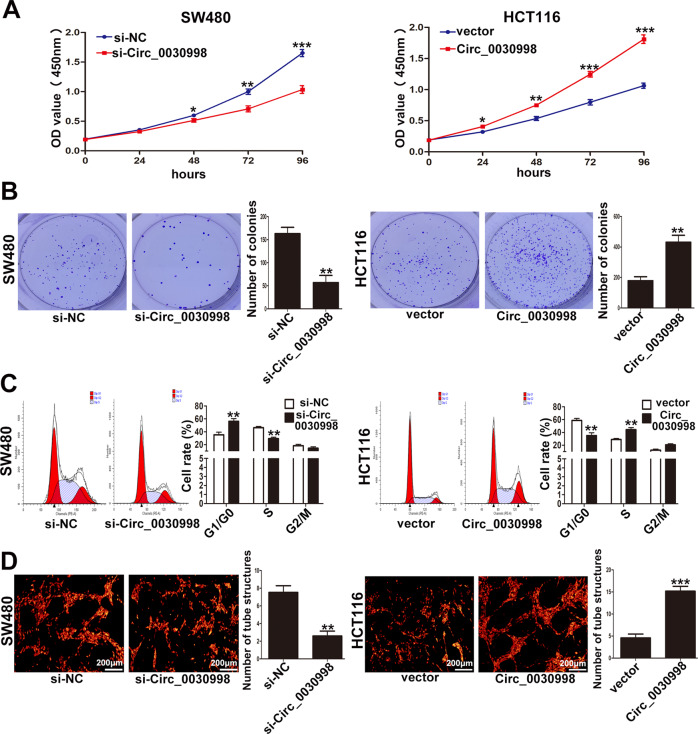
Circ_0030998 promoted CRC cell proliferation and angiogenesis in vitro. **A** The proliferation ability of SW480 cells transfected with si-Circ_0030998 and HCT116 cells transfected with Circ_0030998 plasmid determined by CCK8 assays. **B** The cloning ability of transfected SW480 and HCT116 cells. **C** The effect of Circ_0030998 on cell cycle of SW480 and HCT116 cells. **D** The tube-like structures formation of HUVECs cultured in Matrigel-coated plates with conditioned medium from SW480 or HCT116 cells. *p < 0.05,**p < 0.01, ***p < 0.001.

Then, RNA fluorescence in situ hybridization assays with specific Cy3-labeled probes for Circ_0030998 were performed to identify the subcellular localization of Circ_0030998, and the results demonstrated that Circ_0030998 was mainly localized in the cytoplasm of SW480 and HCT116 cells (Fig. 2D). Next, CCK-8 and colony formation assays showed that downregulation of Circ_0030998 significantly inhibited the proliferation and the cloning ability of SW480 cells; whereas, overexpression of Circ_0030998 promoted these functions in HCT116 cells (Fig. 3A, B). Moreover, flow cytometry analyses showed that Circ_0030998 downregulation led to a significant G1/G0 phase arrest in SW480 cells and vice versa in HCT116 cells when Circ_0030998 was overexpressed (Fig. 3C). Furthermore, HUVECs were used to examine the effect of Circ_0030998 on angiogenesis, and the results showed that the formation of tube-like structures was significantly inhibited when Circ_0030998 was downregulated and vice versa when Circ_0030998 was overexpressed (Fig. 3D). These results suggested that Circ_0030998 acted as an oncogene that promoted the CRC cell proliferation and angiogenesis.

### Downregulation of Circ_0030998 inhibited CRC growth in vivo

**Fig. 4:**
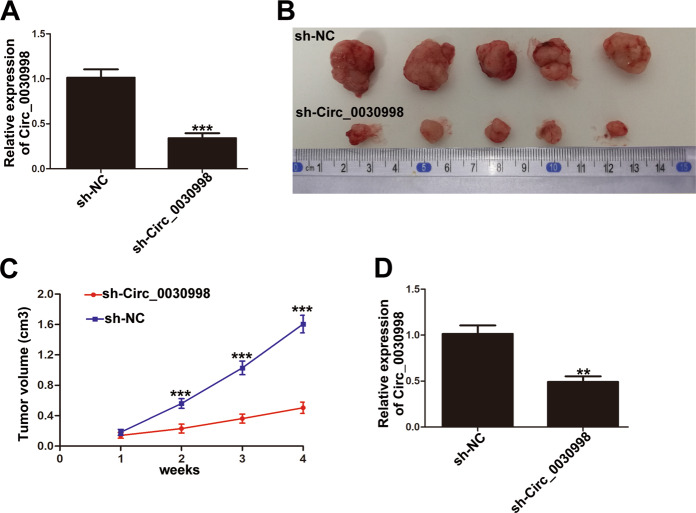
The effect of Circ_0030998 on CRC growth in vivo. **A** The efficiency of sh-Circ_0030998 in SW480 cells examined by qRT-PCR. **B** The tumor tissues from nude mice of SW480/sh-NC and SW480/sh-Circ_0030998 groups. **C** The tumor volumes in two groups were evaluated every week until 4 weeks. **D** The relative expression of Circ_0030998 in tumor tissues from two groups detected by qRT-PCR. **p < 0.01, ***p < 0.001.

To further confirm the roles of Circ_0030998 on tumorigenesis in vivo, shRNA targeting Circ_0030998 was constructed, and the significant knockdown efficiency of sh-Circ_0030998 was verified by qRT-PCR as shown in Fig. 4A. Then, SW480 cells transfected with either sh-NC or sh-Circ_0030998 were injected subcutaneously in the left flank of nude mice. As shown in Fig. 4B and C, the tumor volumes in the sh-Circ_0030998 group were obviously smaller than those in the sh-NC group. Meanwhile, the Circ_0030998 expression in tissues from the sh-Circ_0030998 group was significantly lower than that from the sh-NC group (Fig. 4D). These data suggested that knockdown of Circ_0030998 could inhibit CRC cell proliferation in vivo.

### Circ_0030998 facilitated CRC cell proliferation and angiogenesis by sponging miR-567

**Fig. 5:**
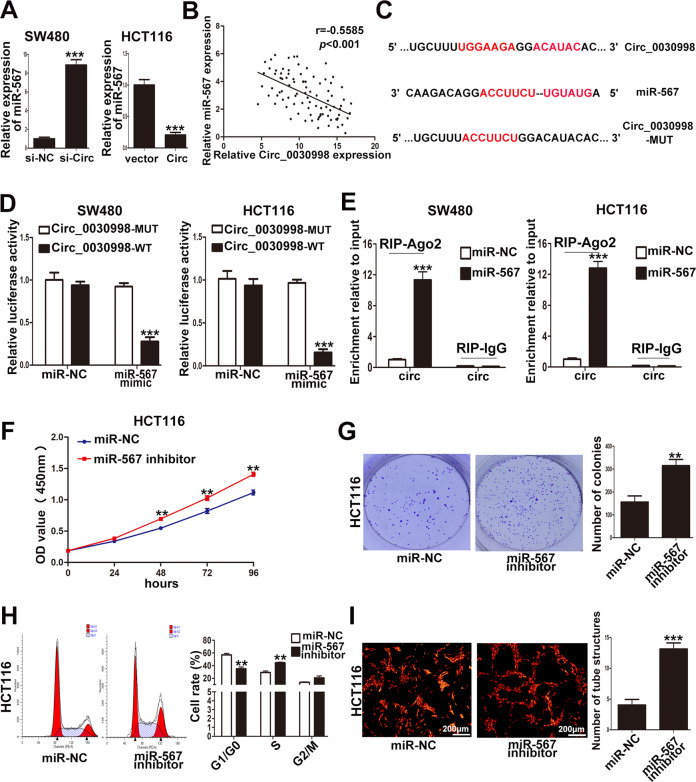
MiR-567 was a target of Circ_0030998 and inhibited the progression of CRC. **A** The expression of miR-567 in SW480 cells when Circ_0030998 was knockdown and HCT116 cells when Circ_0030998 was overexpressed. **B** The inverse correlation between miR-567 and Circ_0030998 in CRC tissues. **C** The predicted binding sites of miR-567 and Circ_0030998. **D** The results of the luciferase reporter assay validated the interaction between miR-567 and Circ_0030998. **E** The level of Circ_0030998 enriched by RIP was detected in SW480 and HCT116 cells transfected with miR-567 or miR-NC. **F** The effect of miR-567 on HCT116 cell proliferation determined by CCK8 assays. **G** The cloning ability of HCT116 cells transfected with miR-567 inhibitor. **H** The effect of miR-567 on cell cycle of HCT116 cells. **I** The tube-like structures formation of HUVECs cultured with conditioned medium from HCT116 cells. **p < 0.01, ***p < 0.001.

Many studies have revealed that circRNAs functioned as “miRNA sponges”, namely, competing endogenous RNAs (ceRNAs), to block the formation of Ago2-mediated silencing complex. Because Circ_0030998 was mainly localized in the cytoplasm of CRC cells, we supposed that Circ_0030998 may also function as a ceRNA. Then two bioinformatics databases, circBank (http://www.circbank.cn/index.html) and Circular RNA Interactome (https://circinteractome.nia.nih.gov/), were used to screen out miRNAs that could bind with Circ_0030998. As shown in Table S3, only miR-567 and miR-556-5p were both predicted by two databases. QRT-PCR showed that only miR-567 was upregulated in SW480 cells when Circ_0030998 was knockdown and downregulated in HCT116 cells when Circ_0030998 was overexpressed (Fig. 5A). Furthermore, we examined miR-567 expression in 90 CRC tissues, and a significant inverse correlation was found between miR-567 and Circ_0030998 as shown in Fig. 5B; so we hypothesized that miR-567 was the miRNA sponged by Circ_0030998.

To further validate whether Circ_0030998 could interact with miR-567 directly in CRC cells, the wildtype and mutated putative binding sites of Circ_0030998 were cloned and inserted into luciferase reporter vectors respectively for luciferase reporter assays (Fig. 5C). The results showed that the luciferase activity of the wild-type but not mutant Circ_0030998 was significantly inhibited by miR-567 mimics in both SW480 and HCT116 cells (Fig. 5D). Furthermore, the RIP assays demonstrated that miR-567 significantly increased the enrichment of Circ_0030998 by Ago2 RIP in both SW480 and HCT116 cells compared to miR-NC (Fig. 5E). These results suggested that Circ_0030998 functioned as a ceRNA for miR-567 in CRC cells.

Next, we examined the effect of miR-567 on CRC cell proliferation and angiogenesis. CCK-8 and colony formation assays showed that downregulation of miR-567 by miR-567 inhibitor increased HCT116 cell proliferation (Fig. 5F, G). Flow cytometry analyses demonstrated that miR-567 inhibitor promoted HCT116 cell cycle progression (Fig. 5H). Moreover, inhibition of miR-567 increased the tube-like structures formation of HUVECs (Fig. 5I).

**Fig. 6:**
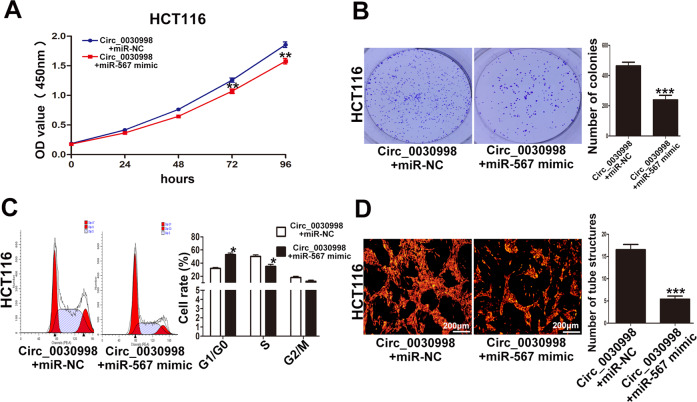
Circ_0030998 regulated CRC cell proliferation and angiogenesis via miR-567. **A** The proliferation ability of HCT116 cells cotransfected with Circ_0030998 plasmid and miR-567 mimic. **B** The cloning ability of cotransfected HCT116 cells. **C** The cell cycle of cotransfected HCT116 cells. **D** The tube-like structures formation of HUVECs cultured with conditioned medium from HCT116 cells. *p < 0.05,**p < 0.01, ***p < 0.001.

Furthermore, we performed rescue assays to confirm whether Circ_0030998 regulated CRC cell proliferation and angiogenesis via miR-567. CCK-8 and colony formation assays showed that miR-567 mimic could partially weaken the promotive effect of Circ_0030998 on HCT116 cell proliferation (Fig. 6A, B). MiR-567 mimic also reversed the promotive effect of Circ_0030998 on HCT116 cell cycle progression and tube-like structures formation of HUVECs (Fig. 6C, D). Taken together, these results suggested that Circ_0030998 promoted CRC cell proliferation and angiogenesis via miR-567.

### MiR-567 inhibited CRC cell proliferation and angiogenesis via VEGFA

**Fig. 7:**
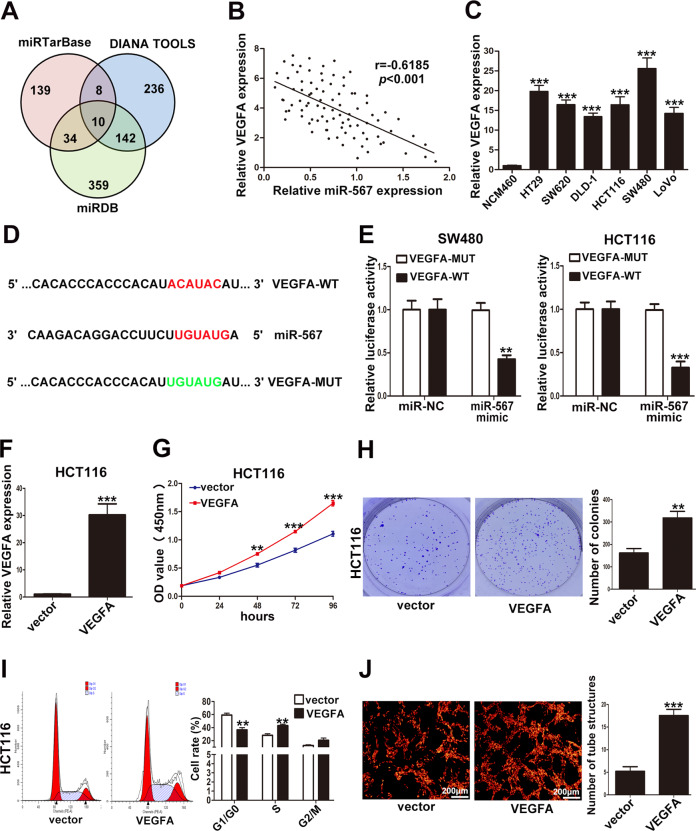
VEGFA was the target of miR-567 and promoted CRC cell proliferation and angiogenesis. **A** The potential target genes of miR-567 predicted by three databases. **B** The inverse correlation between miR-567 and VEGFA in CRC tissues. **C** Relative expression of VEGFA in CRC cell lines and human non-tumorigenic colorectal epithelial cell line NCM460 determined by qRT-PCR. **D** The predicted binding sites of miR-567 and VEGFA. **E** The results of the luciferase reporter assay validated the interaction between miR-567 and VEGFA. **F** The efficiency of VEGFA plasmid in HCT116 cells examined by qRT-PCR. **G** The effect of VEGFA on HCT116 cell proliferation determined by CCK8 assays. **H** The effect of VEGFA on the cloning ability of HCT116 cells. **I** The effect of VEGFA on the cell cycle of HCT116 cells. **J** The tube-like structure formation of HUVECs cultured with conditioned medium from HCT116 cells. **p < 0.01, ***p < 0.001.

MiRNAs could regulate target mRNAs by binding with their 3′UTRs via complementary base pairing. Previous studies showed that miR-567 could regulate KPNA4 [21], ATG5 [22] and then inhibited tumor progression or chemoresistance. To explore the mechanism by which miR-567 inhibited CRC cell proliferation and angiogenesis, three miRNA databases were used to predict the potential target genes in the present study. As shown in Fig. 7A and Table S4, there were 10 potential target genes in the overlapped fraction of three databases, namely BNC2, BVES, CSRNP3, KANSL1L, MB21D2, MBNL2, NEUROD2, UBR3, VEGFA, ZSWIM6. Among these 10 candidate genes, only VEGFA could promote cancer proliferation and angiogenesis. So we hypothesized that VEGFA may be the downstream target gene of miR-567 in regulating CRC cell proliferation and angiogenesis.

Then, we examined miR-567 and VEGFA expression in 90 CRC tissues, and it showed that VEGFA expression was negatively correlated with miR-567 in CRC tissues (Fig. 7B). We also examined the expression of VEGFA in six CRC cell lines and NCM460 by qRT-PCR, it showed that VEGFA was significantly upregulated in CRC cell lines than NCM460 (Fig. 7C). To further confirm the interaction between miR-567 and VEGFA, we performed luciferase reporter assays with luciferase reporter vectors containing the wild-type or mutated putative binding sites of VEGFA (Fig. 7D). The results showed that the luciferase activity of the wild-type VEGFA was significantly inhibited by miR-567 mimics in both SW480 and HCT116 cells compared with mutant VEGFA (Fig. 7E).

Moreover, we designed pcDNA-VEGFA for ectopic expression to verify the effect of VEGFA on CRC cell proliferation and angiogenesis. The efficiency of pcDNA-VEGFA was examined by qRT-PCR as shown in Fig. 7F. CCK-8 and colony formation assays showed that overexpression of VEGFA promoted HCT116 cell proliferation (Fig. 7G, H). Flow cytometry analyses demonstrated that VEGFA promoted HCT116 cell cycle progression (Fig. 7I). Meanwhile, VEGFA promoted the tube-like structures formation of HUVECs (Fig. 7J). The effect of VEGFA on HCT116 cells and HUVECs coincided with that of miR-567 inhibitor. These findings suggested that miR-567 inhibited CRC cell proliferation and angiogenesis via VEGFA.

### Circ_0030998 promoted CRC cell proliferation and angiogenesis via the miR-567/VEGFA axis

**Fig. 8:**
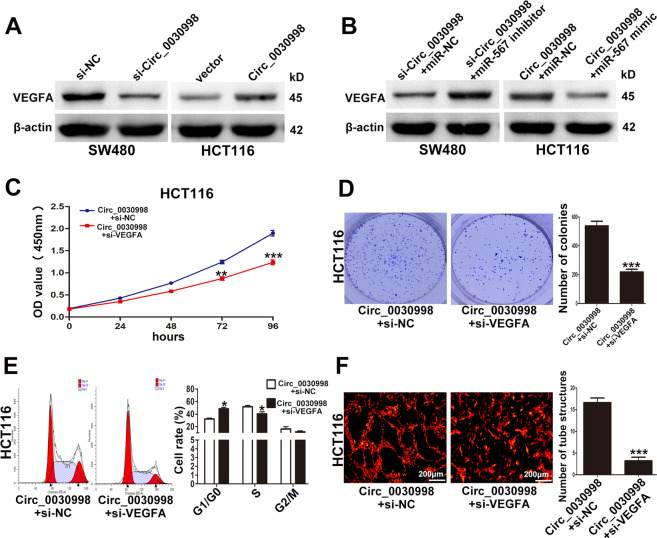
Circ_0030998 promoted CRC cell proliferation and angiogenesis relying on VEGFA. **A** The effect of Circ_0030998 on the expression of VEGFA in CRC cells examined by western blotting. **B** The role of miR-567 in the regulation of Circ_0030998 on VEGFA in CRC cell examined by western blotting. **C** The proliferation ability of HCT116 cells cotransfected with Circ_0030998 plasmid and si-VEGFA. **D** The cloning ability of cotransfected HCT116 cells. **E** The cell cycle of cotransfected HCT116 cells. **F** The tube-like structures formation of HUVECs cultured with conditioned medium from HCT116 cells. *p < 0.05,**p < 0.01, ***p < 0.001.

Furthermore, western blotting showed that VEGFA was decreased in SW480 cells transfected with si-Circ_0030998, and vice versa in HCT116 cells when Circ_0030998 was overexpressed (Fig. 8A). Moreover, miR-567 inhibitor or mimic could partially reverse the effect of Circ_0030998 on VEGFA in SW480 and HCT116 cells (Fig. 8B). We also designed si-VEGFA and conducted rescue assays to confirm whether Circ_0030998 functioned in CRC via VEGFA. CCK-8 and colony formation assays showed that the effect of Circ_0030998 on HCT116 cell proliferation was partially reversed by si-VEGFA (Fig. 8C, D). Si-VEGFA also weakened the promotive effect of Circ_0030998 on HCT116 cell cycle progression and tube-like structures formation of HUVECs (Fig. 8E, F). All these data suggested that Circ_0030998 promoted CRC cell proliferation and angiogenesis via the miR-567/VEGFA axis.

## Discussion

Till now, more than 100,000 human circRNAs have been discovered [23, 24], and many of them have been detected playing important roles in various physiological and pathological processes such as cell differentiation [25], cell cycle progression [26], cellular proliferation [27], apoptosis of cancer cells [28], immune tolerance [29] and so on. Several circRNAs have been validated effective in the progression of CRC [16–18]. In the present study, we analyzed the microarray data GSE138589, which compared differentially expressed circRNAs in six pairs of CRC tissues and matched neighboring normal tissues, and confirmed a new circRNA, Circ_0030998, was upregulated in CRC tissues. The dysregulation of Circ_0030998 was related to CRC patients’ survival, suggesting its potential diagnostic and therapeutic value as a biomarker. Moreover, Circ_0030998 significantly regulated CRC cell proliferation in vitro and in vivo, and also affected the tube formation of HUVECs.

CircRNAs are formed by back-splicing of primary transcripts in the nucleus and then transported to the cytoplasm via ATP-dependent RNA helicase DDX39A and spliceosome RNA helicase DDX39B [30]. RNA fluorescence in situ hybridization was an effective method for the visualization of circRNA. In the present study, we identified that Circ_0030998 was mainly localized in the cytoplasm of CRC cells by FISH, which indicated that Circ_0030998 may function as a miRNA sponge. Accumulating studies have demonstrated that circRNAs in the cytoplasm mainly played their roles as competing endogenous RNAs [31, 32]. MiR-567 was predicted by bioinformatics analysis and further validated by luciferase reporter assay as the target miRNA of Circ_0030998 in our study. Furthermore, a negative correlation between miR-567 and Circ_0030998 was detected in CRC tissues. All these data suggested that Circ_0030998 served as a ceRNA in the regulation of CRC proliferation and angiogenesis.

MicroRNAs (miRNAs), approximately 21–23nt in length, mainly play a post-transcriptional regulatory role in gene expression. The mature miRNAs in cytoplasm bind with Argonaute protein family members and then the miRNA-induced silencing complex (miRISC) forms [33]. This complex binds to the 3′ untranslated region of the target mRNA through miRNA according to the principle of base pairing, thereby exerting an inhibitory or silencing effect [34, 35]. MiR-567, a tumor suppressor gene, has been studied in several cancers. In breast cancer, miR-567 could inhibit cancer cell proliferation and migration by regulating KPNA4 [21]; miR-567 could also regulate autophagy and reverse trastuzumab resistance via ATG5 in breast cancer [22]; in renal cell carcinoma, miR-567 could inhibit cancer cells progression by regulating PRDX3 [36]. Consistent with the reported studies, we found that inhibition of miR-567 could promote CRC cell proliferation and angiogenesis. Moreover, overexpression of miR-567 could partially reverse the effect of Circ_0030998 on CRC cells and HUVECs. Next, we predicted the target mRNA of miR-567 by three bioinformatic algorithms (miRTarBase, miRDB, and DIANA TOOLS), and confirmed that VEGFA was a target of miR-567 by luciferase reporter assay.

Angiogenesis is an important factor in solid tumor progression, and anti-angiogenesis has been demonstrated effective in cancer therapy [37]. VEGFA is a primary factor driving the tumor vascular formation [38, 39]. In tumor tissues, a variety of cells can produce VEGFA, such as cancer cells, endothelial cells, and tumor-associated macrophages [40]. VEGFA mainly functions by binding to the receptors, especially VEGFR2, and activates the VEGFR2-dependent signaling pathways [41]. Moreover, VEGFA can accelerate tumor progression by promoting EMT and metastasis [42]. In patients with colorectal cancer liver metastasis, VEGFA could be a prognostic biomarker [43]. In the present study, VEGFA was predicted and validated to be the target of miR-567. Overexpression of VEGFA promoted CRC cell proliferation and cell cycle progression, induced the tube-like structure formation of HUVECs. Furthermore, VEGFA was found to be in positive correlation with Circ_0030998 in CRC cells. Rescue assays demonstrated that the effects of Circ_0030998 on CRC cell proliferation and tube formation of HUVECs could be partially reversed by VEGFA downregulation. Altogether, our data suggested that Circ_0030998 functioned in CRC proliferation and angiogenesis by regulating VEGFA via miR-567.

In conclusion, the present study identified the high expression of Circ_0030998 in CRC tissues and cell lines, revealed the relationship between Circ_0030998 and clinicopathologic features, the prognosis of CRC patients. Moreover, Circ_0030998 served as a ceRNA for miR-567 and then relieved the inhibitory effect of miR-567 on VEGFA, resulting in the proliferation and angiogenesis of CRC eventually. Our findings demonstrated the Circ_0030998/miR-567/VEGFA regulation axis in CRC and suggested that Circ_0030998 could be a potential therapeutic target for CRC.

## Material and methods

### Collection of clinical data and tissue specimens

A total of 90 paired CRC tissues and neighboring nontumor tissues were obtained from patients diagnosed as CRC after surgery in Shanghai General Hospital (Shanghai Jiaotong University School of Medicine, Shanghai, China) from 2010 to 2014. None of the patients received any chemotherapy or radiotherapy before the surgery. Complete clinicopathological data were collected from every patient. All tissues were stored in liquid nitrogen before RNA extraction. The study was approved by the Human Ethics Committee of Shanghai General Hospital, and every patient signed the informed consent.

### Cell culture

All the human CRC cell lines (HT29, SW620, DLD-1, HCT116, SW480, LoVo) and a human non-tumorigenic colorectal epithelial cell line (NCM460) were purchased from the Cell Bank of the Chinese Academy of Science (Shanghai, China). McCoy’s 5A complete medium (Gibco, USA) was used for the culture of HT29, HCT116; RPMI-1640 medium (Gibco, USA) was used for the culture of SW620, DLD-1, LoVo, NCM460, SW480. All the cells were cultured in the medium containing 10% fetal bovine serum (Gibco, USA) at 37 °C with 5% CO_2_.

### QRT-PCR assay

RNA was extracted from tissues or cells with TRIzol reagent (Invitrogen, USA), then Primer-Script One Step RT-PCR kit (TaKaRa, China) was used for reverse transcription and SYBR Premix Dimmer Eraser kit (TaKaRa, China) was used for RT-PCR. All the primers used in the present study were designed by Shanghai Sangon Biotech Co.Ltd and shown in Table S1. GAPDH was used for normalization and the relative expression fold changes of RNAs were calculated with the 2^-ΔΔCt^ method. All the assays were performed in triplicate.

### RNA fluorescence in situ hybridization (RNA-FISH)

Cy3-labeled probe for Circ_0030998 or U6 was designed and generated by RiboBio (Guangzhou, China) and Fluorescent in Situ Hybridization kit (RiboBio, China) was used for the identification of the subcellular localization of Circ_0030998 according to the manufacturer’s recommendations. After co-staining with DAPI for the cell nuclei, the images were acquired. All the assays were performed in triplicate.

### Cell transfection

The sequences of small interference RNAs for Circ_0030998, VEGFA, and scrambled negative control (NC) siRNAs were designed and synthesized by GenePharma (Shanghai, China). MiR-567mimics, miR-567 inhibitors, and miR-NC were purchased from Shanghai Sangon Biotech Co.Ltd. Circ_0030998 and VEGFA cDNA were amplified and cloned into pCDH and pcDNA3.0 vector respectively for ectopic overexpression. The small hairpin RNA (shRNA) of Circ_0030998 was designed and synthesized from GenePharma (Shanghai, China). QRT-PCR was conducted to examine the amplification and knockdown efficiencies. The sequences of siRNAs and shRNA used in the present study were shown in Table S1.

### Cell counting kit-8 (CCK-8) assay

The ability of CRC cell proliferation was examined using CCK-8 kit (Beyotime, China). A total of 1 × 10^3^ transfected CRC cells were seeded into each well of a 96-well plate, and the absorbance at 450 nm was measured every 24 h for 96 h. Each group was cultured in five replicate wells and all assays were conducted in triplicate.

### Colony formation assay

A total of 200 transfected CRC cells were seeded into each well of a six-well plate. After incubation for two weeks, the cells were fixed with 4% paraformaldehyde and stained with 0.1% crystal violet. Then the numbers of the colonies were counted by visual inspection and all the assays were conducted in triplicate.

### Flow cytometric analysis

All the transfected CRC cells were harvested after incubation for 48 h. Then the cells were fixed in pre-chilled 70% ethanol for 16 h at 4 °C and Cell Cycle Analysis Kit (Beyotime, China) was used to stain cells for 30 min with propidium iodide according to the protocol. Flow cytometry (BD Biosciences, USA) was used for cell cycle analysis at last and all the assays were performed in triplicate.

### Tube formation assay

Red fluorescent proteins (RFP) expressing human umbilical vein endothelial cells (HUVECs) were obtained from Angio-Proteomie (Boston, USA) and cultured in endothelial growth medium (EGM-2) and passage 5 was used for the experiments. 50 μl of precooled Matrigel (BD, Corning, USA) was coated into each well of a 96-well plate and polymerized for 30 min at 37 °C. Next, 2 × 10^4^ HUVEC-RFP cells were suspended in a mixture of conditioned medium (50 μl) and endothelial cell medium (50 μl) containing 10% FBS. The fluorescence detection of tube formation was photographed using inverted microscopy (IX-53, Olympus) after 6 h of incubation at 37 °C.

### Bioinformatics analysis

Two bioinformatics databases, circBank (http://www.circbank.cn/index.html) and Circular RNA Interactome (https://circinteractome.nia.nih.gov/), were used to screen for miRNAs that could bind with Circ_0030998. Three miRNA databases, miRTarBase (http://mirtarbase.mbc.nctu.edu.tw/php/index.php), DIANA TOOLS (http://diana.imis.athena-innovation.gr/DianaTools/index.php) and miRDB (http://www.mirdb.org/), were used to predict the potential target genes of miR-567 in the present study.

### Luciferase reporter assay

The putative binding sequences and mutant sequences of miR-567 in Circ_0030998 and VEGFA 3′-UTR were amplified and inserted into pGL3-promoter vector to generate luciferase reporter constructs (GenePharma, China). Then the CRC cells were co-transfected with luciferase reporter constructs and miR-567mimics or miR-NC. After incubation for 48 h, the renilla and firefly luciferase activities were measured by the Dual-Luciferase Reporter Assay System (Promega), and all the assays were performed in triplicate.

### RNA immunoprecipitation (RIP)

HCT116 and SW480 cells transfected with miR-567 mimic or miR-NC were lysed in RIP lysis buffer. The Magna RNA immunoprecipitation kit (Millipore, USA) was used to examine the combination of Circ_0030998 and miR-567 according to the manufacturer’s protocol. The magnetic beads pre-coated with antibodies against Ago2 or IgG were used for precipitation. QRT-PCR was performed to quantify the level of Circ_0030998 enriched by RIP and all the assays were performed in triplicate.

### Western blotting

RIPA lysis buffer and protease inhibitor cocktail were used for the extraction of total proteins from cells. BCA Protein Assay Kit (Beyotime, China) was used to determine the protein concentrations. Then the protein lysates were separated by SDS-PAGE and transferred to PVDF membranes. After incubation with primary antibodies at 4 °C overnight, the membranes were incubated with the corresponding secondary antibodies at room temperature for 1 h. Finally, the proteins were evaluated by chemiluminescence according to the manufacturer’s instructions. Antibodies used in the present study were: VEGFA (26381-1-AP, Proteintech), β-actin (60008-1-Ig, Proteintech), LAMP1(21997-1-AP, Proteintech). All assays were performed in triplicate.

### Animal experiment

Four-week-old male athymic BALB/c nude mice were used in the present study. Ten mice were randomly divided into two groups according to the random number table, and injected subcutaneously with SW480 cells (100 μl, 1 × 10^6^) transfected with either sh-NC or sh-Circ_0030998 in the left flank. Then, all the mice were maintained under specific-pathogen-free conditions and the tumor volumes were measured as 0.5 × length × width^2^ weekly. Four weeks later, all the mice were sacrificed and all the tumors were excised. The researcher was blinded to the group allocation during the assays. The study was approved by the Animal Care and Use Committee of Shanghai General Hospital.

### Statistical analysis

All the continuous data were shown as mean ± standard deviation. Student’s t-test or One-way ANOVA was applied to compare group differences. χ^2^ test was used to evaluate the correlation between clinicopathologic characters and Circ_0030998 expression. For survival analysis, the Kaplan-Meier method with the log-rank test was conducted. Univariate and multivariate Cox proportional hazards models were performed to analyze the prognostic factors. All the statistical analyses were completed using SPSS 20.0 and a two-sided *p*-value less than 0.05 was considered statistically significant.

## Supplementary information

Table S1

Table S2

Table S3

Table S4

## Data Availability

All data presented in the study is available from the corresponding author on reasonable request.
